# Spontaneous regression of adrenal metastasis from renal cell carcinoma after sunitinib withdrawal: case report and literature review

**DOI:** 10.1186/s12894-018-0420-x

**Published:** 2018-11-14

**Authors:** Ti-Yuan Yang, Wun-Rong Lin, Allen W. Chiu

**Affiliations:** 1 Department of Urology, Mackay Memorial Hospital, Taipei, Taiwan; 2 Department of Medicine, Mackay Medical College, Taipei, Taiwan; 3 School of Medicine, National Yang-Ming University, Taipei, Taiwan

## Abstract

**Background:**

The spontaneous regression of metastatic renal cell carcinoma is a rare phenomenon, with an estimated incidence of < 1%. We report a case of post-nephrectomy renal cell carcinoma adrenal metastasis, followed by the spontaneous regression of the metastasis after withdrawal of sunitinib.

**Case presentation:**

The patient was a 55-year-old male with clear cell type renal cell carcinoma who previously underwent a left laparoscopic radical nephrectomy. After 51 months of follow up, a recurrence in the left renal fossa was observed and subsequently excised. Four months after excision, an abdominal Computerized tomography (CT) identified an adrenal metastasis of 1.6 cm. The patient was treated with sunitinib. However, the treatment was discontinued because of gastrointestinal side effects and fatigue. Eleven months after the discontinuation of sunitinib treatment, a progression in the adrenal metastasis growth (5.7 cm) was observed, whereas 16 months after the discontinuation, a regression of the adrenal metastasis growth (3.4 cm) was observed. During subsequent follow-ups, a gradual reduction in the size of the adrenal metastasis (1.8 cm) was observed. After 44 months from the discontinuation of sunitinib treatment, the patient was still alive and followed up in the outpatient department.

**Conclusions:**

Sunitinib is a multi-targeted inhibitor of vascular endothelial growth factor (VEGF) receptors. This compound reduces tumor angiogenesis and has been approved worldwide for the treatment of advanced renal cell carcinoma. To our knowledge, this is the fourth case of the spontaneous regression of metastatic renal cell carcinoma after the discontinuation of sunitinib treatment.

## Background

Approximately 21% of patients with renal cell carcinoma present with a metastatic disease at diagnosis, and 23% of patients who undergo radical nephrectomy for clinically localized disease develop metastasis/local recurrence during a 5-year follow-up [1]. The spontaneous regression of metastatic renal cell carcinoma is a rare but well-known phenomenon, with an estimated incidence of < 1% [2]. Several case reports have described the spontaneous regression of metastatic renal cell carcinoma. Such an occurrence has been associated with multiple different events that might influence the immune system, including primary tumor surgical debulking, radiation or embolization of the primary tumor, palliative hormonal treatment with tamoxifen, surgical abortion, and discontinuation of sunitinib treatment [3–6]. However, the exact mechanism remains unclear. We report a case of a post-nephrectomy adrenal metastasis of a renal cell carcinoma followed by the spontaneous regression of the metastasis after a short-term sunitinib treatment. To our knowledge, this is the fourth case of the spontaneous regression of metastatic renal cell carcinoma after withdrawal of sunitinib.

## Case presentation

**Fig. 1:**
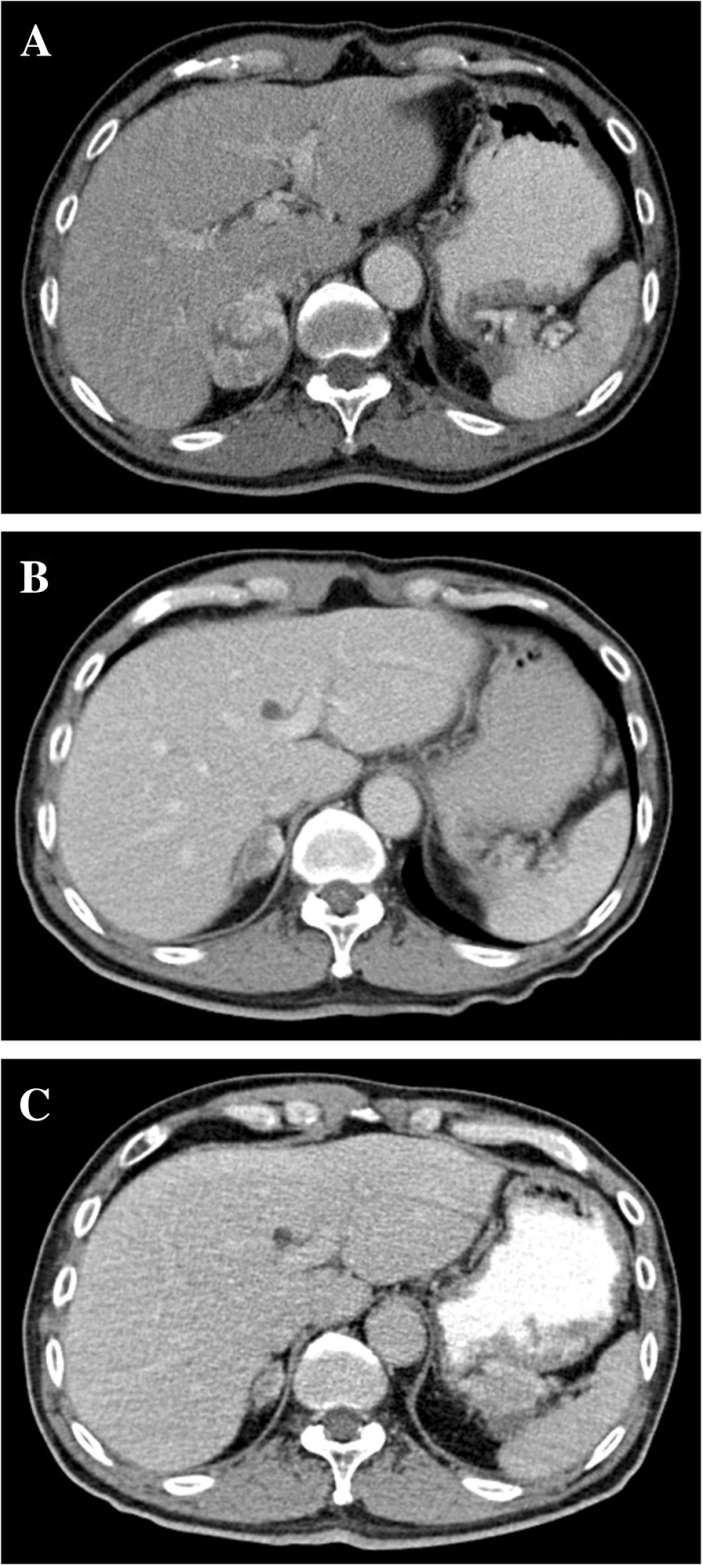
Abdominal CT examination revealed spontaneous regression of adrenal metastasis (left to right). **a**. Adrenal metastasis measuring 5.7 cm in diameter. **b**. Adrenal metastasis measuring 3.4 cm in diameter. **c**. Adrenal metastasis measuring 1.8 cm in diameter

A 55-year-old man presented with chronic testicular pain. An ultrasonography of the abdomen detected left renal tumor. The patient had a history of hypertension and left renal urolithiasis. CT showed a heterogeneous left upper pole renal tumor (5.3 cm in diameter). A laparoscopic radical nephrectomy was performed in May 2008. Left adrenalectomy and lymph node dissection were not performed because the CT scan showed no adrenal gland invasion or lymphadenopathy. The histological evaluation of the tissue revealed a clear cell renal cell carcinoma and negative surgical margins (pathological stage, T2N0M0). Three years after nephrectomy, following a cerebrovascular accident, the Eastern Cooperative Oncology Group score changed from 0 to 2. No tumor recurrence (CT scan was performed every 6 months) was found until 51 months later. A CT scan detected two nodules in the renal fossa (1.8 and 0.9 cm, respectively). Retroperitoneal exploration confirmed recurrent clear cell carcinoma with microscopically positive surgical margins. Lymph node dissection was not performed because of severe adhesion around the aorta. Lymph nodes that could be detected by palpation were not identified during the surgery. Four months after excision, an abdominal CT showed a nodule (1.6 cm) over the right adrenal gland. At that time, tumor target therapy was not covered by the national health insurance in Taiwan. Therefore, because of economic reasons, the patient could not afford the treatment until 2013. A repeat CT evaluation confirmed the disease progression of the adrenal metastasis (2.1 cm). The patient was treated with sunitinib (37.5 mg/d) for 4 weeks, but the treatment was discontinued because of gastrointestinal side effects and fatigue. After 3 months, a CT scan showed the progression of the adrenal metastasis (3.8 cm) and no lower lung lesion. A chest X-ray revealed the absence of lung metastasis. The patient refused to undergo hormonal survey, biopsy, and adrenalectomy. Eleven months after sunitinib treatment, a CT scan showed an obvious growth of the adrenal metastasis (5.7 cm) (Fig. 1a), whereas 16 months after the treatment, a regression of the metastasis (3.4 cm) was observed (Fig. 1b). Twenty-two months after sunitinib treatment, a CT scan demonstrated a gradual reduction in the size of the adrenal metastasis (1.8 cm) (Fig. 1c). The patient was still alive and followed up at the outpatient department 44 months after the discontinuation of sunitinib treatment.

## Discussion and conclusions

The spontaneous regression of cancer is defined as the partial or complete disappearance of a tumor without any treatment or with a treatment considered inadequate to exert a significant influence on the progression of cancer [7]. The spontaneous regression of metastatic renal cell carcinoma following nephrectomy was first described by Bumpus in 1928 [8]. It is a rare phenomenon, which is estimated to represent < 1% of renal cell carcinoma cases [9]. The regression of metastatic sites has been reported to occur at the lungs and at other visceral organs including liver, bones, brain, choroid, pancreas, and adrenal glands [10]. The mechanism of the spontaneous regression of renal cell carcinoma remains unclear. Humoral, immunological, and vascular factors, such as autoinfraction, have been previously proposed to be possible pathophysiologic mechanisms [11]. Nephrectomy is not necessary and accounts for < 50% of the documented cases [12]. Because the oncologic benefits of lymph node dissection in the management of renal cell carcinoma remain controversial [13], we routinely performed nephrectomy and renal fossa recurrent tumor resection without lymph node dissection.

Sunitinib is a multi-targeted inhibitor of vascular endothelial growth factor (VEGF) receptors. The mechanism of action includes the reduction of tumor angiogenesis, which makes it an approved treatment for advanced renal cell carcinoma worldwide [14]. It has been shown that response rates are comparable among groups treated with 50 mg/d of sunitinib for 4 weeks followed by 2 weeks off treatment or with 37.5 mg/d of sunitinib on a continuous daily dosing treatment [15]. A study performed on 1059 patients treated with sunitinib for metastatic renal cell carcinoma has shown that 398 (38%) patients had an objective response and 12 (1.1%) had a complete response. The median time to tumor response was 10.6 weeks [16]. Our report describes the case of a patient who experienced metastasis progression after discontinuation of sunitinib treatment because of adverse effects on day 28 and spontaneous regression 16 months later. To our knowledge, there are only three other reports that have described a similar phenomenon. Rothermundt et al. reported a 63-year-old female patient who had a right renal tumor with the invasion of the right renal vein and the vena cava, bilateral adrenal metastasis, multiple lung metastases, and bone metastasis [17]. The patient was treated with 50 mg of sunitinib 4 weeks on and 2 weeks of. A decrease in the size of all involved tumor sites was observed at the beginning of treatment. After 10 months, because an obvious disease was detected on CT, sunitinib treatment was discontinued. A CT scan performed for trial purposes after 1 month revealed disease regression in all tumor locations. Yanagihara et al. were the second to report a case of the regression of metastatic renal cancer after the discontinuation of sunitinib treatment [18]. The authors reported the case of a 61-year-old female patient with a left renal tumor along with multiple bone and liver metastases. Left radical nephrectomy revealed disease progression in each metastatic site. The patient was treated with sunitinib, but the treatment was discontinued on day 11 because of thrombocytopenia and digestive symptoms. Disease regression was detected after 18 months. Teo et al. reported the case of a 65-year-old male patient [6] with lung metastasis, which was detected by CT scan 7 years after a right radical nephrectomy was performed for right renal cell carcinoma. Sunitinib was started for the metastasis. A partial response was achieved 9 months after starting the treatment; however, the treatment was discontinued because of disease progression after 6 months, which was followed by the regression of lung metastasis. The patient remains clinically well with a follow up of 44 months since the discontinuation of sunitinib treatment. A summary of the published cases of the discontinuation of sunitinib treatment is listed in Table 1. Data from three published cases and those from our patient showed that the interval of sunitinib treatment varied from 11 days to 15 months. The interval between spontaneous regression and the last sunitinib dose varied from 1 to 18 months. Two cases discontinued sunitinib treatment because of disease progression and two discontinued it because of the side effects of the drug.

**Table 1:** Summary of published sunitinib withdrawal phenomenon cases

Ref, year	Age/sex	Location	Dose and length of sunitinib treatment	Reason for discontinuing sunitinib treatment	Interval between spontaneous regression and sunitinib
Rothermundt, 2009 [17]	63/F	bilateral adrenal glands, lung and bone	sunitinib 50 mg 4 weeks on and 2 weeks off, 10 months	disease progression	1 month
Yanagihara, 2011 [18]	61/F	bone and liver	sunitinib 50 mg per day, 11 days	thrombocytopenia and digestive symptoms	18 months
Teo, 2013 [6]	65/M	lung	50 mg/d, 4 weeks on and 2 weeks off for 6 months and 37.5 mg per day for 9 months	disease progression	No mention

The mechanisms of spontaneous regression after an incomplete use of the multiple kinase inhibitor sunitinib remain unclear. Rothermundt et al. drew an analogy with the antiandrogen withdrawal syndrome of prostate cancer. Gene mutations of the androgen receptor might be a possible mechanism of antiandrogen withdrawal syndrome, which cause the antiandrogens to act as partial agonists. A withdrawal of these antiandrogens can promote disease regression [19].

Another possible mechanism is the immunomodulatory effect of sunitinib. Sunitinib improved the type-1 T-cell cytokine response in patients with metastatic renal cell carcinoma while reducing the T-regulatory cell function [20]. Furthermore, sunitinib has been shown to inhibit the proliferation and function of human peripheral T cells and to prevent T-cell-mediated immune response in mice [21]. In the present report, sunitinib treatment, or its discontinuation, might have modified the immune response.

The phenomenon of spontaneous regression after the discontinuation of sunitinib treatment may be masked by further treatment. The disease regression might have been missed or attributed to other second line therapies if our patient had not refused the suggestion of a right adrenalectomy. Thus, the number of patients with spontaneous regression associated with the discontinuation of sunitinib treatment may be underestimated, and this case report is a reminder of that for urologists.

Because recurrence has been reported after spontaneous regression [5], we will closely follow up our patient.
